# p62 Pathology Model in the Rat Substantia Nigra with Filamentous Inclusions and Progressive Neurodegeneration

**DOI:** 10.1371/journal.pone.0169291

**Published:** 2017-01-11

**Authors:** Kasey L. Jackson, Wen-Lang Lin, Sumitra Miriyala, Robert D. Dayton, Manikandan Panchatcharam, Kevin J. McCarthy, Monica Castanedes-Casey, Dennis W. Dickson, Ronald L. Klein

**Affiliations:** 1 Department of Pharmacology, Toxicology, and Neuroscience, Louisiana State University Health Sciences Center, Shreveport, LA, United States of America; 2 Department of Neuroscience, Mayo Clinic, Jacksonville, FL, United States of America; 3 Department of Cell Biology and Anatomy, Louisiana State University Health Sciences Center, Shreveport, LA, United States of America; 4 Department of Pathology, Louisiana State University Health Sciences Center, Shreveport, LA, United States of America; Niigata Daigaku, JAPAN

## Abstract

One of the proteins most frequently found in neuropathological lesions is the ubiquitin binding protein p62 (sequestosome 1). Post-mortem analysis of p62 is a defining diagnostic marker in several neurodegenerative diseases including amyotrophic lateral sclerosis and inclusion body myositis. Since p62 functions in protein degradation pathways including autophagy, the build-up of p62-positive inclusions suggests defects in protein clearance. p62 was expressed unilaterally in the rat substantia nigra with an adeno-associated virus vector (AAV9) in order to study p62 neuropathology. Inclusions formed within neurons from several days to several weeks after gene transfer. By electron microscopy, the inclusions were found to contain packed 10 nm thick filaments, and mitochondria cristae structure was disrupted, resulting in the formation of empty spaces. In corollary cell culture transfections, p62 clearly impaired mitochondrial function. To probe for potential effects on macroautophagy, we co-expressed p62 with a double fluorescent tagged reporter for the autophagosome protein LC3 in the rat. p62 induced a dramatic and specific dissociation of the two tags. By 12 weeks, a rotational behavior phenotype manifested, consistent with a significant loss of dopaminergic neurons analyzed post-mortem. p62 overexpression resulted in a progressive and robust pathology model with neuronal inclusions and neurodegeneration. p62 gene transfer could be a novel methodological probe to disrupt mitochondrial function or autophagy in the brain and other tissues in vivo.

## Introduction

Neurodegenerative diseases are typically characterized by specific diagnostic protein inclusions, and the inclusions often include the protein p62 (sequestosome 1)[1–3]. p62 is involved in protein trafficking and protein degradation, both in the macroautophagy pathway and the ubiquitin-proteasome system [4,5]. p62 recognizes polyubiquitinated substrate proteins and traffics them for degradation, and p62 itself is a substrate for autophagic degradation [6]. Thus, compromised protein degradation could lead to the build-up of both p62 and its cargo proteins in the cell.

Mutations in p62 may result in several degenerative diseases with p62 inclusions [7–12]. However, p62 is commonly found in neuropathological inclusions even when it is not mutated in sporadic disease forms [3, 9,13,14]. It is this much more common, non-mutated p62 inclusion pathology in sporadic disease forms that we attempted to recapitulate using a vector for human wild-type p62 in this study. p62 pathological aggregates are found co-localized with ubiquitin and alpha-synuclein in the Lewy bodies of Parkinson’s disease [1, 13], which involves degeneration of the substantia nigra. We expressed p62 using a recombinant adeno-associated virus (AAV9) in the nigrostriatal pathway of the rat as a model for p62-induced neurodegeneration.

We hypothesized that when p62 was overexpressed, neuropathological inclusions would form leading to neurodegeneration/neuronal loss. We attempted to determine inclusions by light microscopy for p62 and the related protein degradation proteins, ubiquitin and ubiquilin-2 [3] and by electron microscopy. p62 is known to bind microtubule-associated protein 1 light chain 3 (LC3) and function as a chaperone in autophagy [6]. We investigated for potential effects on macroautophagy by co-expressing p62 with a double fluorescent tagged form of LC3 [5]. Castillo et al. (2013) utilized AAV gene transfer of double-tagged LC3 to track the progression of autophagy [15]. Upon combining this autophagy reporter with the autophagy-related protein p62, we hypothesized that p62 would induce dissociation of the two fluorophores and the formation of red-only puncta consistent with the progression of LC3 to the autolysosome. Since effects on autophagy could exert effects on mitochondria we also tested whether p62 overexpression would alter mitochondrial structure in vivo and mitochondrial function in transfected cells.

## Materials and Methods

### DNA and AAVs

cDNA for human wild-type p62 (SC117669 from Origene) was incorporated into an AAV expression cassette plasmid described in Klein et al. (2002) [16]. The cassette has AAV2 terminal repeats, the hybrid cytomegalovirus/chicken β-actin promoter, the woodchuck hepatitis virus post-transcriptional regulatory element, and the bovine growth hormone polyadenylation sequence. We used the same cassette to separately express either green fluorescent protein (GFP) or a double-tagged EGFP/mCherry LC3B (Addgene plasmid #22418 by J. Debnath) [17]. A transgene-less (Empty) version was used as a control in cell culture experiments.

DNAs were packaged into recombinant AAV9 as previously described [18]. Helper and AAV9 capsid plasmids used to generate AAV9 were from the University of Pennsylvania [19]. Viral stocks were sterilized using Millipore Millex-GV syringe filter, aliquoted, and stored frozen. Viral genome copies were titered using dot-blot assay, and equal titer doses were obtained by diluting stocks in lactated Ringer’s solution from Baxter Healthcare.

### Animals and vector injections

A total of 23 young, adult female Sprague-Dawley rats (approximately 12 weeks old and weighing about 225 g) received unilateral injections to the substantia nigra as described [20]. The stereotaxic injection coordinates for the substantia nigra were 5.3 mm posterior to Bregma, 2.1 mm lateral, and 7.6 mm ventral. The vector dose used for stereotaxic injections of either AAV9 p62 (N = 14) or AAV9 GFP (N = 4) was 2.2 x 10^10^ vector genomes (vg) diluted in a volume of 3 μl. We expressed p62 on a unilateral basis so that we could track side-to-side changes relative to the contralateral uninjected side and because the degree of neuronal loss can be monitored by rotational behavior [18, 20, 21]. The contralateral side was left uninjected and used as an internal reference control for non-transduced, untreated tissue. These animals were studied between 5 days and 12 weeks after gene transfer in histological assays. In some rats, the same dose of the AAV9 p62 was combined with AAV9 double-tagged LC3 (N = 3). The AAV9 double-tagged LC3 was co-applied at a dose of 1 x 10^10^ vg. As a control for the effects of p62 on LC3, we expressed AAV9 double-tagged LC3 (at the same dose) by itself or combined with AAV9 GFP (dose of 2.2 x 10^10^ vg).

All animal care and procedures were in accordance with the Animal Care and Use Committee at Louisiana State University Health Sciences Center at Shreveport and NIH guidelines. The Animal Care and Use Committee at Louisiana State University Health Sciences Center at Shreveport approved this study. Animals were housed 2 per cage, maintained on a 12 light-dark cycle, and given water and PicoLab Rodent Diet 20 (5053) *ad libitum*. The rats were monitored twice weekly for signs of pain or distress; however, no rats became severely ill or died prior to the endpoint of the study. Steps were taken to ameliorate pain and suffering, and animals were administered a cocktail of anesthetics including ketamine, xylazine, and acepromazine prior to euthanasia. Euthanasia consisted of either transcardial perfusion or decapitation.

### Immunofluorescence and immunohistochemistry

Animals were anesthetized and perfused with phosphate-buffered saline (PBS) followed by cold 4% paraformaldehyde (PFA) in PBS as previously described [20]. Tissues were removed and immersed in 4% PFA overnight at 4°C followed by cryoprotection in 30% sucrose/PBS. 50 μm sections were cut on a sliding microtome with a freezing stage. Primary antibodies and dilutions are in S1 Table. Secondary antibodies include biotinylated antibodies from DAKO Cytomation (1:2,000), Alexa Fluor 488, and Alexa Fluor 594 (Invitrogen) at 1:300. 4',6-diamidino-2-phenylindole (Sigma) counterstaining followed standard methods. Samples were analyzed at intervals between 5 day and 4 weeks after gene transfer as indicated and at 12 weeks after gene transfer. For immunohistochemistry, animals were perfused transcardially with 4% PFA in PBS. Coronal slices containing the substantia nigra were processed for paraffin embedding. Five μm sections were pre-treated in a steamer with deionized water before immunostained in an autostainer (DAKO, Carpinteria, CA) using 3,3’-diaminobenzidine as the chromogen.

### Electron microscopy

Animals were perfused transcardially with 2.5% glutaraldehyde-2% PFA in 0.1M cacodylate buffer. The substantia nigra was cut into small pieces and post-fixed in 0.1% osmium tetroxide, en bloc stained in 1% uranyl acetate-50% ethanol, dehydrated in alcohols and propylene oxide, infiltrated and embedded in Epon 812. Thin sections were stained with uranyl acetate and lead citrate and examined with a Philips 208S electron microscope fitted with a Gatan 831 Orius CCD digital camera. Digital micrographs were processed with Adobe Photoshop CS5 (64 bit) software.

### Western blotting

Tissues were dissected from the animals and frozen on dry ice. The samples were Dounce-homogenized in RIPA buffer (1% nonidet-P40/0.5% sodium deoxycholate/0.1% sodium dodecyl sulfate/PBS) with protease inhibitors (Halt protease inhibitor cocktail kit from Pierce) and then centrifuged. Protein content was determined by Bio-Rad Protein Assay Dye. Samples were normalized for protein content and electrophoresed in 12% polyacrylamide containing sodium dodecyl sulfate (Bio-Rad). Primary antibodies and dilutions are in S1 Table. Secondary antibodies and ECL reagents were from Santa Cruz.

### Stereological estimates

The number of substantia nigra pars compacta neurons expressing tyrosine hydroxylase immunoreactivity was estimated by unbiased stereology using the MicroBrightfield Inc. system as previously described [20]. Eight sections evenly spaced throughout the pars compacta structure were analyzed for each probe. Optical dissectors were 50 x 50 x 16 μm cubes spaced in a systematic random manner 150 x 150 μm apart and offset 2 μm from the section surface. The fractionator sampling was optimized to yield about 150 counted cells per animal for Gundersen error coefficients <0.10.

### Cell size estimates

Two sections spaced 300 μm apart in the substantia nigra were stained for tyrosine hydroxylase as above, and the substantia nigra pars compacta was photomicrographed with a 10X lens. Using the Scion imaging program, we conducted a particle size estimate for the stained neurons. By using the appropriate size cutoffs, we selectively counted the single cells and avoided clusters of overlapping cells. Approximately 150 cells over the two sections were analyzed for their area size in square pixels. The average cell sizes were calculated, and the cells on the injected side were compared to the uninjected contralateral sides.

### Rotational behavior

Animals were challenged with *d*-amphetamine (free base, 2 mg/kg in saline, intramuscular, Sigma) at 1, 6, and 12 weeks after gene transfer. The amphetamine was injected 20 min before placing the animals in an automated rotometer system from San Diego Instruments for 10 min.

### Cell transfections and mitochondrial bioenergetics measurements

Human embryonic kidney 293T cells were seeded at 15,000 cells/well into Seahorse Bioscience XF microplates, and grown to ~50% confluency. The cells were transfected with plasmid DNA (Empty, GFP, or p62) using the calcium-phosphate method. Each well received 60 ng DNA.

To determine effects of p62 on mitochondrial function, a Seahorse Bioscience XF24 Extracellular Flux Analyzer was used to measure oxygen consumption rates (OCR). The XF24 creates a transient, 7 μL chamber in specialized microplates, which allows determination of oxygen and proton concentrations in real time. Each XF24-assay well contains a disposable sensor cartridge, embedded with 24 pairs of fluorescent biosensors (oxygen and pH), coupled to fiber-optic wave guides. The wave guides deliver light at various excitation wavelengths (oxygen = 532 nm, pH = 470 nm) and transmits a fluorescent signal (oxygen = 650 nm, pH = 530 nm) to a set of photodetectors. Oxygen consumption rate (OCR) was expressed in pMoles/mg protein in control and p62 transfected cells. Cells were cultured in DMEM media for the basal measurements, then treated with oligomycin (1 μg/ml), FCCP (300 nM), and rotenone (10 μM), and the OCR was measured. The ATP-linked OCR was calculated as the basal OCR minus the OCR measured after the addition of oligomycin. The reserve capacity is the FCCP OCR minus the basal OCR.

Glycolysis parameters were also evaluated. For extracellular acidification rate (ECAR) measurements, cells were changed to assay media lacking glucose. Basal ECAR was measured and plotted as a function of cells under the basal condition followed by the sequential addition of glucose (25 mM), oligomycin (1 μg/ml), and 2-deoxyglucose (25 mM). The rate of glycolysis was determined by subtracting the basal ECAR from the ECAR after the addition of glucose. Glycolytic reserve was determined by subtracting the ECAR following the addition of oligomycin from the ECAR following the addition of glucose. ECAR was expressed in mPH/mg protein. Measurements were made in triplicate from 3 independent samples. OCR and ECAR measurements were made 2 and 3 days after transfection, respectively. The methods for oxygen consumption and glycolysis have been described previously in Liu et al. (2014) [22].

### Statistical methods

Statistical analyses were performed on GraphPad Prism 5.0. Statistical tests included paired t-tests, non-paired t-tests, and one-way analysis of variance (ANOVA) with Bonferroni post-tests as indicated. Data are expressed as mean ± standard error of the mean.

## Results

### Hypertrophy and then loss of dopaminergic neurons

**Fig 1:**
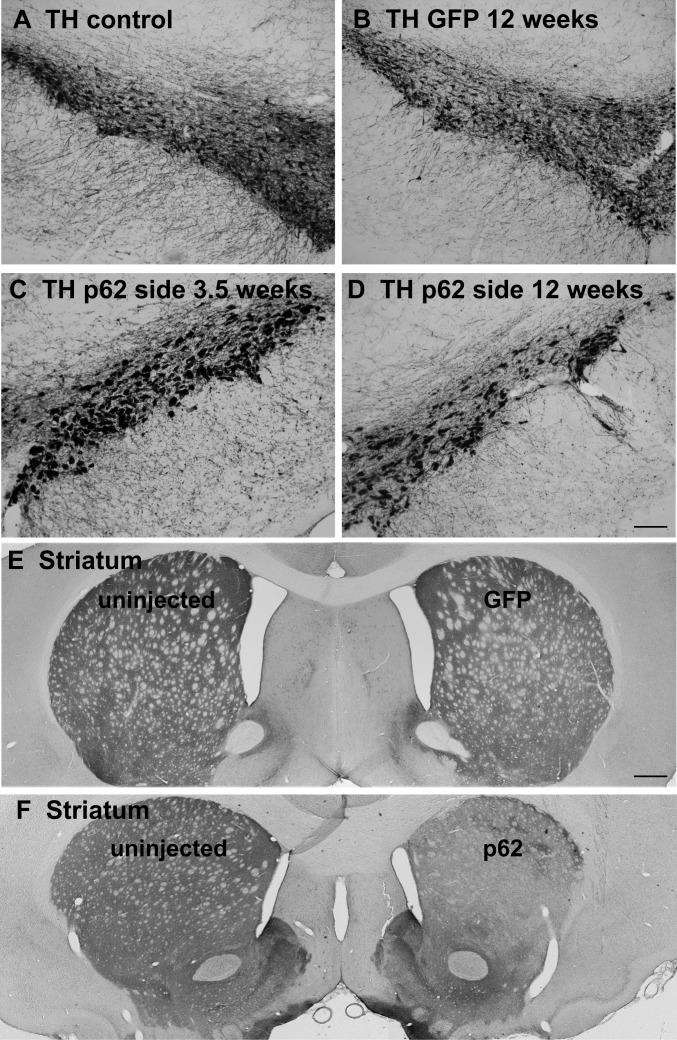
Tyrosine hydroxylase staining of dopaminergic neurons in the nigrostriatal pathway. A) Substantia nigra from an uninjected side of the brain. B) Substantia nigra from an AAV9 GFP injected side after 12 weeks after gene transfer. C) Substantia nigra from an AAV9 p62 injected side at 3.5 weeks after gene transfer. D) Substantia nigra from an AAV9 p62 injected side at 12 weeks after gene transfer. There is a noticeable hypertrophy of the neurons on the p62 side at both intervals yet an apparently progressive loss of cells between the two time points in the p62 group. E) Forebrain from a GFP animal at 12 weeks after gene transfer. F) Forebrain from a p62 animal at 12 weeks after gene transfer. There is a loss of striatal tyrosine hydroxylase on the side where AAV9 p62 was injected into the substantia nigra. Bar in D = 134 μm; same magnification in A-C. Bar in E = 536 μm; same magnification in F.

By 12 days after gene transfer, p62 was significantly increased on the injected side compared to the contralateral uninjected side by western blot (S1 Fig, P < 0.01, paired t-test, N = 3/side). At longer intervals after gene transfer, 3.5 and 12 weeks, we noticed a striking hypertrophy of neurons (enlargement of perikarya) stained for tyrosine hydroxylase on the side of the AAV9 p62 injections (Fig 1C and 1D). The clearly noticeable hypertrophy was observed both at 3.5 and 12 weeks, the longest interval studied. We quantified the tyrosine hydroxylase positive neuronal sizes at 12 weeks and found a 41% enlargement of the cells on the p62 side compared to the uninjected side (S2 Fig, P < 0.05, paired t-test, N = 3/side). In contrast, the control AAV9 GFP vector did not alter cell size (S2 Fig). In the AAV9 p62 injected substantia nigra, along with the increase in cell size, there was a progressive loss of tyrosine hydroxylase neurons (Fig 1C and 1D). There appeared to be greater loss of tyrosine hydroxylase immunoreactivity at 12 weeks relative to 3.5 weeks, although we did not quantify cell profiles at 3.5 weeks to make a statistical comparison between the two intervals. Analysis of the tyrosine hydroxylase positive neurons in the substantia nigra at 12 weeks indicated a significant cell loss in the p62 group. Stereological estimates demonstrated a 48% loss of dopamine neurons on the p62 side relative to the uninjected side (S2 Fig, P < 0.0001, paired t-test, N = 7/side). Consistent with this finding of a lesion to the substantia nigra, the density of tyrosine hydroxylase positive fibers in the striatum was noticeably reduced on the p62 side (Fig 1E and 1F). The unilateral AAV9 GFP injections also resulted in lower numbers of tyrosine hydroxylase stained neurons in the injected substantia nigra compared to the uninjected side by 19% (S2 Fig, P < 0.05, paired t-test, N = 4/side). Partial cell toxicity is not unexpected with efficient GFP gene transfer [23], but in contrast, the lesioning effect of the AAV9 p62 was more pronounced. The percent loss of stained cells was greater in the p62 group compared to the GFP group (S2 Fig, P < 0.005, t-test, N = 4-7/group), consistent with the rotational behavior manifesting in the p62 group but not the GFP group. The signature p62 pattern was qualitatively different from the effect of the GFP, i.e., there was no cell hypertrophy or inclusions in the GFP group. We also stained sections for the neuronal marker NeuN. The NeuN staining pattern consistently reflected lowered number of cells in the substantia nigra pars compacta in both the p62 and GFP transduced samples relative to the uninjected sides (S3 Fig), supporting genuine cell loss in both groups rather than mere loss of tyrosine hydroxylase.

### Rotational behavior

**Fig 2:**
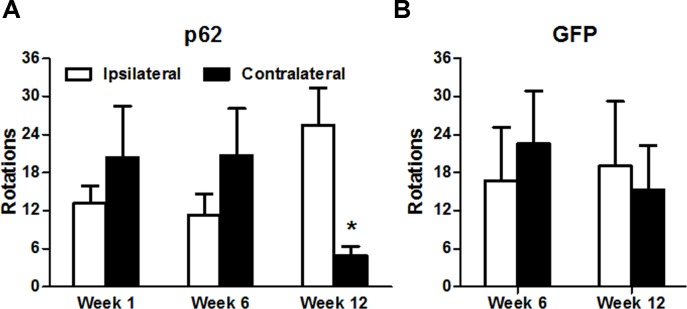
Development of a turning bias over time in rats overexpressing p62 unilaterally in the substantia nigra. Amphetamine-stimulated rotations occur when there is a large side-to-side difference in dopamine levels in the nigrostriatal pathway in rats, i.e., when there is a large loss of dopamine neurons on one side. A) In the p62 group, the behavioral phenotype of ipsilateral turning bias developed by 12 weeks (N = 7, P < 0.05, t-test), but not at earlier times in the p62 group. B) Turning bias did not manifest in a group of rats expressing the control protein GFP (N = 4).

Amphetamine-induced rotational behavior is a behavioral phenotype resulting from the loss of substantia nigra dopaminergic neurons in the rat. As in Parkinson’s disease, a substantial fraction (~50%) of the substantia nigra dopamine neurons must be lost before the rotational bias manifests [21, 24]. Rats circle towards the ipsilateral side of the lesion after amphetamine administration, indicating a major dopamine imbalance on the two sides of the brain [21, 24]. A turning bias developed over time in p62 rats but not control rats (Fig 2). There was no imbalance at 1 and 6 weeks, but by 12 weeks a significant bias had developed in the p62 group. At some point between 6 and 12 weeks there was sufficient loss of dopamine neurons to manifest as a turning bias in p62 transduced rats, but not GFP expressing control rats.

### p62 inclusion formation and ubiquitin and ubiquilin-2 immunoreactivity

**Fig 3:**
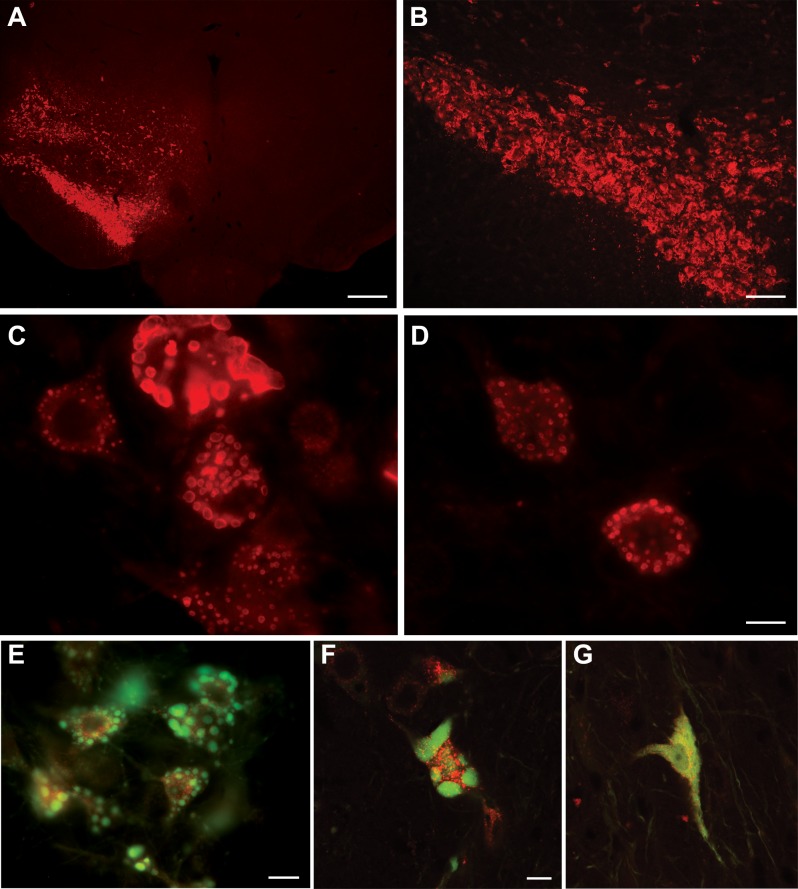
p62 inclusions. A, B) p62 immunoreactivity was induced on one side of the ventral midbrain. White rectangle in (A) shows the area of enlargement in (B). C, D) The overexpression induced numerous inclusions within the transduced neurons. E, F) Visualization of inclusions in p62 expressing cells using co-expression of a green and red double-tagged LC3 reporter protein. The p62 caused severe dissociation of the two tags. G) In a control neuron expressing the LC3 reporter without the p62, the two tags remain together and superimpose. The time point in these samples was 21–25 days. Bar in A = 536 μm; bar in B = 134 μm; bar in D = 14 μm; same magnification in C; bar in E = 14 μm; bar in F = 10 μm; same magnification in G.

**Fig 4:**
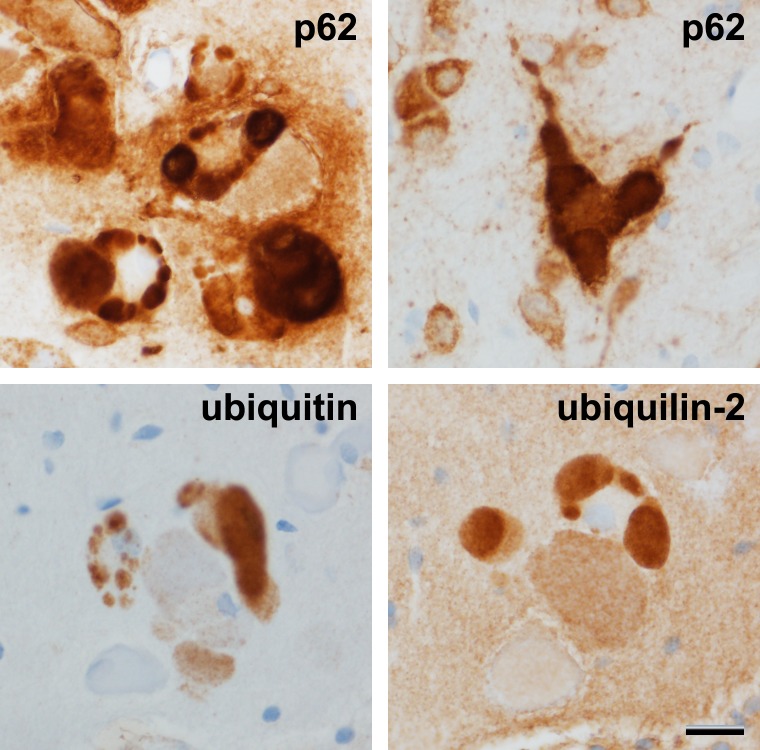
p62-, ubiquitin-, and ubiquilin-2-positive inclusions. p62, ubiquitin, and ubiquilin-2 are involved in protein degradation pathways. A, B) p62-positive inclusions. C, D) ubiquitin- or ubiquilin-2 positive inclusions on adjacent sections (thin paraffin sections). The recombinant p62 expression led to deposition of endogenous rat ubiquitin and ubiquilin-2 into aggregates. The time point was 21 days after gene transfer. Bar in D = 20 μm; same magnification in A-C.

p62 expression was unequivocally elevated on the AAV9 p62 injected side (Fig 3A and 3B). In contrast, control gene transfer with AAV9 GFP did not induce p62 expression relative to the contralateral, uninjected side (not shown). p62-positive inclusions efficiently formed within the transduced neurons (Fig 3C and 3D). Small inclusions formed as early as 5 days, the earliest time point studied. In some animals, we combined AAV9 p62 with AAV9 double-tagged LC3. The fluorophores of the LC3 facilitated visualization of the inclusions: p62 efficiently dissociated the two tags (Fig 3E–3G, S4 Fig). The fluorescent structures were three dimensional and therefore difficult to capture in complete focus on a fluorescent microscope (Fig 3C–3E). Interestingly, on adjacent thin sections, we saw an induction of inclusions specifically labeled with either ubiquitin or ubiquilin 2 (Fig 4). However, we were unable definitively co-localize p62 immunoreactivity with ubiquitin and ubiquilin-2 to the same inclusions on the same sections. We were also unable to detect alpha-synuclein-positive inclusions in the tissues.

**Fig 5:**
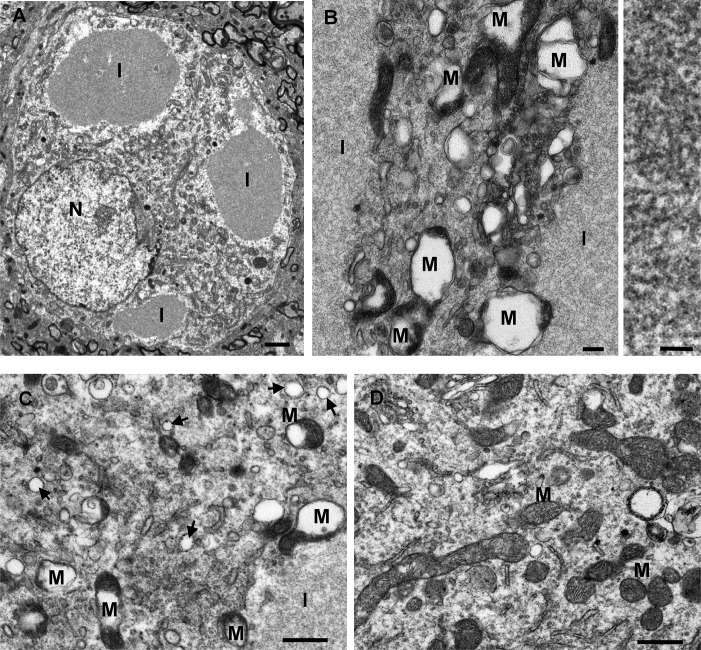
Electron microscopy: filamentous inclusions and disruption of mitochondria induced by p62 gene transfer. A, B) Consistent with the p62-positive inclusions observed by light microscopy, there were numerous inclusions (I) viewed within neurons on the AAV9 p62 injected side, but not found on the uninjected side. The filaments were densely packed in non-membrane bound inclusions and had a width of approximately 10 nm (right panel in B). B, C) In cells with the inclusions, the mitochondria (M) were grossly abnormal with the disruption of cristae structure and the formation of vacuoles within the mitochondria. Arrows point to small vesicles. D) A sample from the uninjected side of the brain shows normal mitochondria. Time point of 9 days after gene transfer in A, and 21 days after gene transfer in B-D. Bar in A = 1 μm; bar in B left = 0.2 μm, bar in B right = 50 nm; bar in C, D = 0.1 μm.

Two time intervals were studied by electron microscopy, 9 and 21 days after gene transfer. The p62-induced inclusions were readily visible and frequent within neurons on the injected side (Fig 5A and 5B). The filaments were densely packed in inclusions and found to be 10 nm (Fig 5B). Though only based on qualitative appearance, at 9 days there were relatively more of the smaller filamentous inclusions, and at 21 days, the inclusions were usually larger in size suggesting progressive enlargement over time. The inclusions were non-membrane bound and often found in close proximity to mitochondria. They were also observed in neuronal dendrites at synapses. The filamentous inclusions were not present on the contralateral uninjected side. The control AAV9 GFP gene transfer did not induce inclusions or structural changes to mitochondria (S5 Fig).

### p62 overexpression disrupts mitochondrial structure in vivo and mitochondrial function in transfected cells

In addition to the inclusions, there was an unequivocal change in the cristae structure of the mitochondria on the injected side. On the AAV9 p62 side, large vacuoles formed within the mitochondria and the regular cristae structure appeared to be torn apart (Fig 5). The structural changes appeared to be more pronounced at 21 days compared to 9 days post-gene transfer, and these mitochondrial disruptions were not found on the uninjected side or in control AAV9 GFP samples (S5 Fig). In addition, small vesicles appeared to be induced in the cytoplasm by the AAV9 p62, as they were more frequently observed on the injected side compared to the uninjected side (Fig 5).

**Fig 6:**
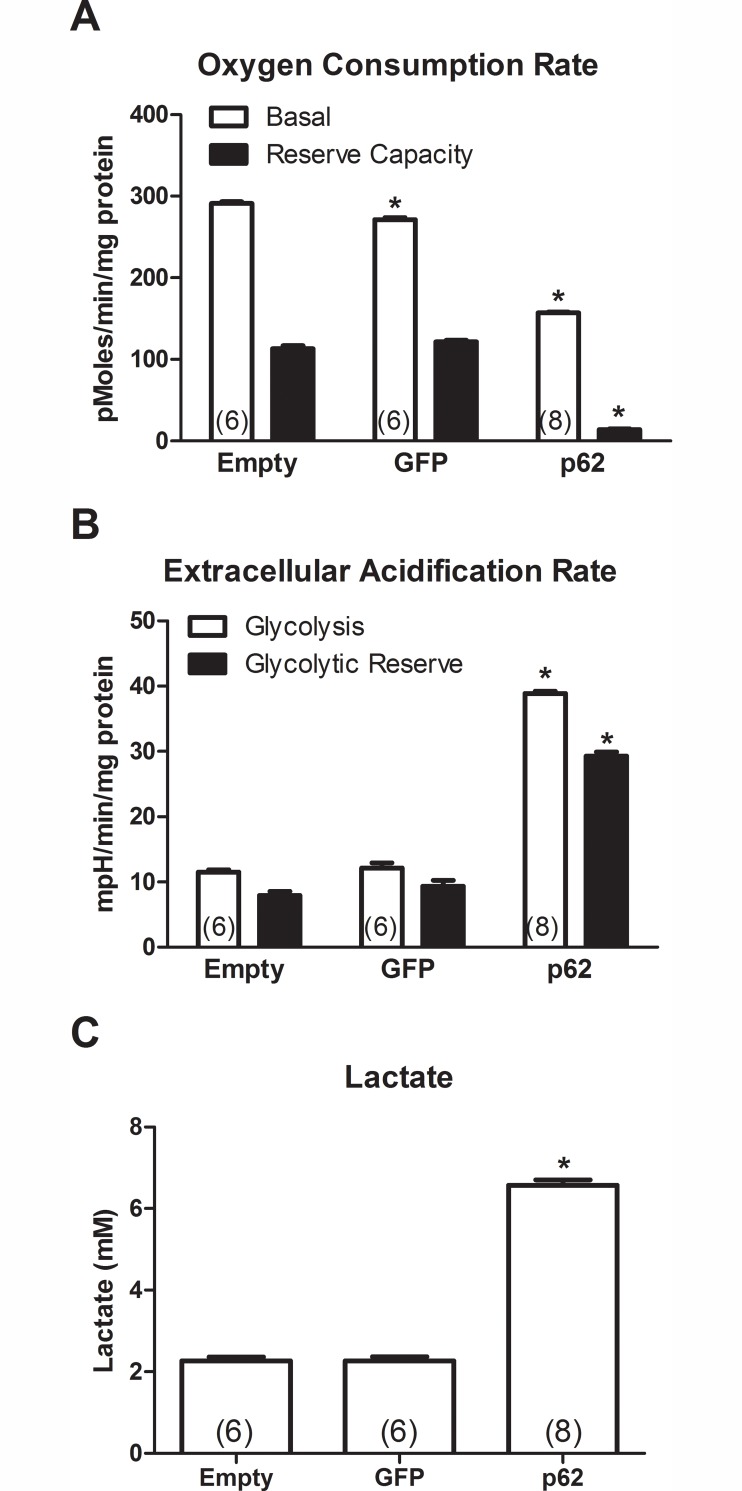
p62 impairs mitochondrial function in transfected cells: decreased oxidative phosphorylation and increased glycolysis. HEK 293T cells were transfected with a plasmid for p62 or control plasmids (GFP and empty). A) Basal oxygen consumption, i.e., oxidative phosphorylation, was decreased in the p62 group compared to the two control groups (ANOVA/Bonferroni, p < 0.001). There was also a small decrease in oxygen consumption in the GFP group relative to the empty group (ANOVA/Bonferroni, p < 0.001). B) Glycolysis and glycolytic reserve were increased in the p62 group compared to the two controls as evaluated by the extracellular acidification rate (ANOVA/Bonferroni, p < 0.001). C) Lactate, a by-product of glycolysis, was increased in the p62 group compared to the two controls (ANOVA/Bonferroni, p < 0.001). N is indicated in parentheses, asterisk indicates significance compared to the empty vector group.

To further study p62’s effect on mitochondria, we assayed oxygen consumption in transfected HEK 293T cells. The basal rate of oxygen consumption in the p62-transfected cultures was clearly decreased relative to the empty vector and GFP control groups (Fig 6A; One-way ANOVA/Bonferroni, p < 0.001). The basal rate of oxygen consumption in the GFP group was also significantly decreased compared to the empty vector group (One-way ANOVA/Bonferroni, p < 0.001) to a lesser degree than in the p62 group. Reserve capacity is the difference in rates before and after the addition of FCCP. FCCP is a mitochondrial uncoupler that opens pores between the matrix and the interspace of the mitochondria, reorganizing the proton gradient. This uncoupling results in continuous transport of protons and a maximal rate of O_2_ consumption. As expected, the FCCP treatment induced an acute increase of the oxygen consumption rate in the two control groups. However, the FCCP-induced increase in the p62 group was severely diminished compared to the two control groups (One-way ANOVA/Bonferroni, p < 0.001) and resulted in total oxygen consumption that was no higher than the basal rate in the two control groups. These results indicate impairment of oxidative phosphorylation and mitochondrial function in the p62-transfected cultures.

We also studied glycolysis in the transfected cells (Fig 6B). The rate of glycolysis was substantially increased in the p62 group compared to the two control groups (One-way ANOVA/Bonferroni, p < 0.001). Glycolytic reserve is the difference between the basal ECAR and ECAR after the addition of oligomycin, i.e. glycolytic capacity. Glycolytic reserve was also increased in the p62 group compared to the two control groups (One-way ANOVA/Bonferroni, p < 0.001). As further confirmation, we measured lactate concentration. Glycolysis generates lactate in the cells, and thus, tissues with high glycolytic activity should show increased concentrations of lactate. The p62 group showed significantly higher lactate concentrations than the two control groups (Fig 6C; One-way ANOVA/Bonferroni, p < 0.001). The data suggest that the intracellular accumulation of p62 affected oxidative phosphorylation and glycolysis.

### p62 drives double-tagged LC3 to the autolysosome on a specific basis

**Fig 7:**
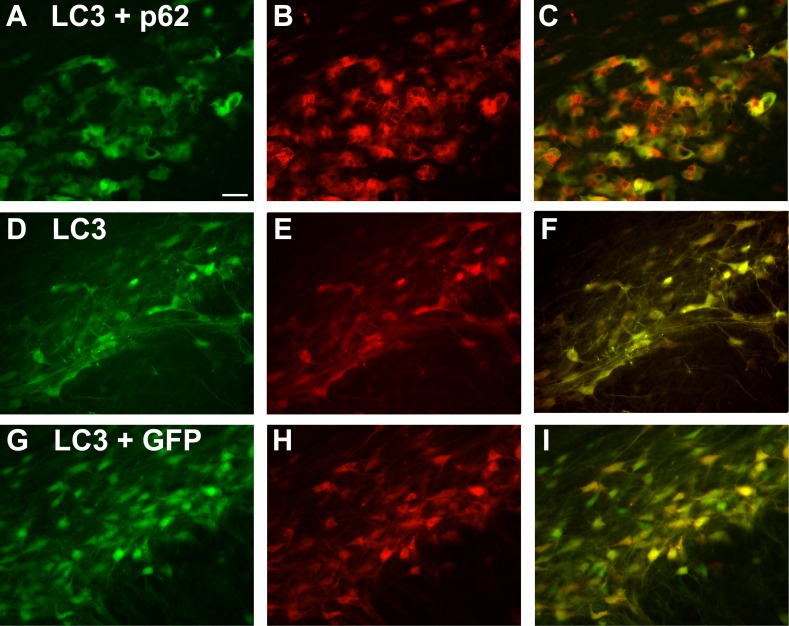
Specific, robust dissociation of the double-tagged LC3 autophagy reporter protein by p62. A green and red fluorescent tagged LC3 was co-expressed with p62, or GFP. The columns from left to right are the green channel, the red channel, and the merger. A-C) With p62, the two tags strongly separate with both red-only and green-only labeling. The formation of the red only puncta (B) is consistent with the progression of the expressed LC3 to the autolysosome. In stark contrast, when LC3 is expressed by itself (D-F) or co-expressed with GFP (G-I), the two tags on the LC3 do not dissociate and superimpose in the merger (yellow). The time point was 22 days after gene transfer. Bar in A = 42 μm. Same magnification in all panels.

To study potential effects on autophagy, we utilized a double-tagged form of LC3 (Fig 7). When AAV9 double-tagged LC3 is expressed by itself, the two fluorophores superimpose (Fig 7D–7F); the LC3 present mostly remains with both fluorophores intact. In stark contrast, when p62 was co-expressed, p62 induced a dramatic dissociation of the two fluorophores with areas within the cell that were dense for small, red-only puncta (Fig 7A–7C) suggesting processing of the expressed LC3 protein to the low pH environment of the autolysosome. Although only a qualitative analysis, the effect of the p62 was dramatic and specific because separation of the two tags did not occur when the LC3 was co-expressed with AAV9 GFP (Fig 7G–7I). We decided the AAV9 GFP vector was an appropriate control to compare with p62 in the LC3 co-expressions since the GFP fluorescence would not interfere with visualization of the red only LC3 puncta in the autolysosomes. The AAV9 GFP did not alter the LC3 pattern compared to when double-tagged LC3 was expressed by itself; the two fluorophores largely superimposed in either case.

## Discussion

Pathological intra-neuronal and intra-myocyte protein inclusions involving p62 are found in a variety of degenerative diseases such as amyotrophic lateral sclerosis (ALS) [7], frontotemporal lobar degeneration [3, 8], inclusion body myositis [9], Paget’s disease of bone [10, 11], and inclusion body myositis with Paget’s disease of bone and frontotemporal lobar degeneration [12]. p62 is also a component of the neurofibrillary tangles of Alzheimer’s disease and other tauopathies as well as the Lewy bodies in Parkinson’s disease [13, 14]. Several neuropathological proteins have been expressed to mimic features of neurodegenerative diseases in diverse animal models. Since p62 pathology is so prevalent in so many types of neuropathologies and because p62 mutations are associated with ALS and inclusion body myositis, we studied whether p62 overexpression would induce pathological lesions and neurodegeneration in rats for the first time. p62 mRNA and protein levels have been shown to be increased in inclusion body myositis [9]. Non-mutated, wild-type p62 is commonly found in sporadic disease forms which were modeled here. The main goal was to establish a p62 neuropathology model with inclusions so that the kinetics of inclusion formation could be studied, and in the future, to study therapeutic interventions that could block the inclusions from forming or deaggregate them. Furthermore, this model has utility for a specific type of protein lesion that can be compared against other specific types of proteinopathies in terms of toxicity and specific responses to treatments. We initially sought to combine p62 expression with transactive response DNA-binding protein, 43 kDa (TDP-43) [25] in order to attempt to precipitate cytoplasmic TDP-43 aggregates, but p62 induced a robust disease state with filamentous cytoplasmic inclusions when expressed by itself. Small p62-positive inclusions began to form as early as 5 days after gene transfer. The inclusions were plentiful in the neuronal perikarya and dendrites and were densely packed with uniform filaments as observed by electron microscopy. The p62 gene transfer caused neuronal hypertrophy which could have been due to the build-up of the inclusions in the cytoplasm. Over time, a behavioral phenotype developed, consistent with the neuronal loss observed post-mortem. The inclusions did not contain alpha-synuclein so the characteristic Lewy bodies of Parkinson’s disease were not achieved. However, the AAV assay system facilitates co-expression experiments as shown here with p62 and LC3, so p62 could be combined with TDP-43, tau, or alpha-synuclein by this method.

Within the transduced cells, the p62 caused severe changes to mitochondria and a specific deposition pattern of an LC3 autophagy reporter protein. The mitochondrial cristae appeared to be shredded apart while large intra-organelle vacuoles formed in the p62 samples. p62 caused a highly specific effect dissociation of the tagged LC3. The red only puncta are consistent with movement of the expressed LC3 into the autolysosome compartment in the p62 samples. The co-expressions of p62 and tagged LC3 in this study were preliminary and the LC3 pattern was not quantified, though p62 clearly induced progression of LC3 to the autolysosome. We cannot conclude that the LC3 progression was concomitant to a net increase in autophagic flux, given the build-up of p62 inclusions and mitopathy, which might suggest impaired macroautophagy. The disruption of the mitochondrial structure in vivo was paralleled by cell culture data demonstrating impaired mitochondrial function in p62-transfected cells. Interestingly, we noted two mildly toxic effects in the GFP control group: slight neuronal loss compared to uninjected tissues and slightly impaired mitochondrial function in transfected cells.

This robust p62 inclusion model could facilitate studies of treatments that disaggregate inclusions made up of p62 or other neuropathological proteins in a specific manner in vivo. Given the various methods of delivering AAV vectors and the tissue-specific targeting that is now possible, the p62 gene transfer could be used to affect autophagy in the CNS or at other specific sites. Several studies have demonstrated that up-regulation of autophagy is potentially beneficial in various models of neurodegenerative diseases [26–28]. With more study, low level or regulated p62 expression could potentially be harnessed to stimulate autophagy leading to the dissolution of toxic neuropathological aggregates and neuroprotection.

## Supporting Information

S1 Table Primary antibodies and dilutions. (DOCX)

S1 Fig p62 overexpression in rat substantia nigra. At a time point of 12 days, the ventral midbrain was dissected and prepared for western blot for p62. Three subjects from the AAV9 p62 group and the three contralateral uninjected sides are shown. The p62 antibody recognized both rat and human p62. Glyceraldehyde 3-phosphate dehydrogenase (GAPDH) was used for normalization.
(TIF)

S2 Fig Analyses of tyrosine hydroxylase immunohistochemistry in the substantia nigra at a 12 week time point. A) The tyrosine hydroxylase positive cells were significantly larger (41%) on the p62 side than the uninjected side (paired t-test, p < 0.05). There was no difference in cell size when AAV9 GFP was administered. B) Cells stained for tyrosine hydroxylase were counted by stereological analysis on the vector injected side and the uninjected side and expressed as a ratio for each animal. There was significant reduction of the tyrosine hydroxylase stained cells in the p62 group compared to the GFP group (t-test, p < 0.005). N values as indicated.
(TIF)

S3 Fig Neuronal staining (NeuN) in the substantia nigra. Compared to uninjected (A, D), there was evidence of a reduction in the number of stained cells in the substantia nigra pars compacta (SNc) in animals administered GFP (B, E) and p62 (C, F). Bar in A is 268 μm; same magnification in B and C. Bar in D is 67 μm; same magnification in E and F.
(TIF)

S4 Fig **Confocal micrographs of neurons expressing double-tagged LC3, either alone (A-C) or with p62 (D-F)**. The p62 increased red-only puncta, consistent with the progression of LC3 to the autolysosome. The merged panels are also shown in Fig 3 with scale bars.
(TIF)

S5 Fig Electron micrographs from a control AAV9 GFP injected rat with 21 day time point. A, B) Neuron in the substantia nigra from the AAV9 GFP injected side. C, D) Neuron in the substantia nigra on the contralateral, uninjected side. B and D are enlargements of A and B, respectively. * in the left panels indicate area of enlargement on the right panels. Cytoplasmic inclusions of any size were absent. The mitochondria (arrows) did not contain vacuoles and there were few vesicles in the cytoplasm in contrast to the AAV9 p62 samples in Fig 5. N, nucleus. A patent vessel in C indicates a successful perfusion. Scale bars (1 μm) are shown in A and C. Scale bar (0.2 μm) is shown in B, same magnification in D.
(TIF)
