# Supplementary material for: p62 Pathology Model in the Rat Substantia Nigra with Filamentous Inclusions and Progressive Neurodegeneration
Source: PLoS One. 2017 Jan 11;12(1):e0169291. doi: 10.1371/journal.pone.0169291 (PMC5226781; doi:10.1371/journal.pone.0169291)
Supplement: S1 Table — (DOCX) [file pone.0169291.s001.docx]

| **Primary Antibody** | **Provider** | **Dilution** | **Application** |
| --- | --- | --- | --- |
| mouse anti-p62 (H00008878-M01) | Abnova | 1:1000 - 1:3000 | WB, IF |
| p62 lck ligand (610832) | BD Biosciences | 1:50000 | IHC |
| rabbit anti-GFP (A11122) | Invitrogen | 1:1000 | IF |
| rabbit anti-tyrosine hydroxylase (P4010-0) | Pel-Freeze | 1:1000 | IF |
| mouse anti-NeuN (MAB377) | Chemicon | 1:500 | IF |
| mouse anti-ubiquitin (Ubi-1; 042691GS) | Millipore | 1:250 - 1:50,000 | IF |
| mouse anti-ubiquilin-2 (H00029978-M03) | Abnova | 1:1000 | IF |
| mouse anti-GAPDH (sc-32233) | Santa Cruz | 1:2000 | WB |
