# Supplementary figures and images for: p62 Pathology Model in the Rat Substantia Nigra with Filamentous Inclusions and Progressive Neurodegeneration
Source: PLoS One. 2017 Jan 11;12(1):e0169291. doi: 10.1371/journal.pone.0169291 (PMC5226781; doi:10.1371/journal.pone.0169291)

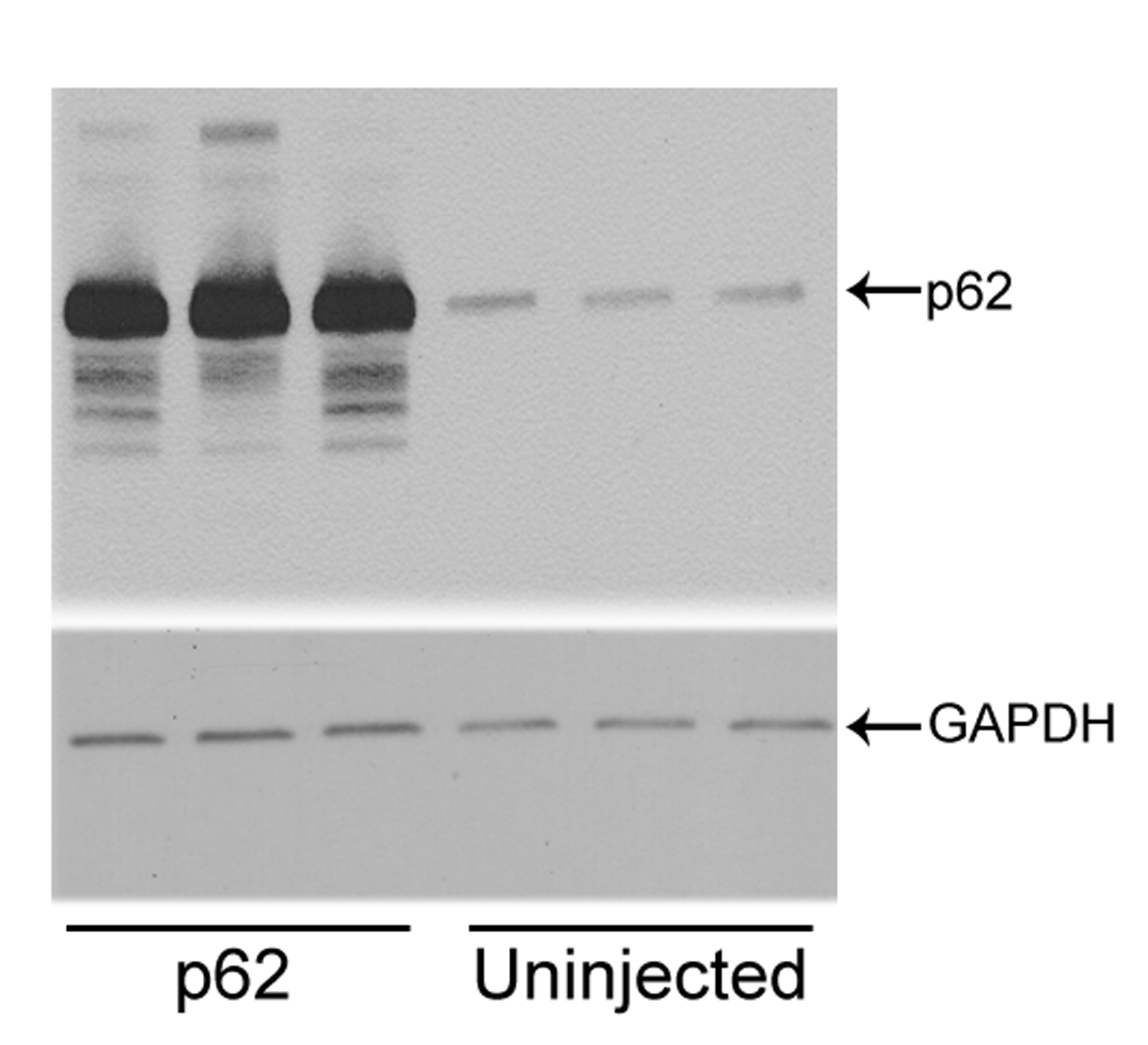

Supplement: S1 Fig — At a time point of 12 days, the ventral midbrain was dissected and prepared for western blot for p62. Three subjects from the AAV9 p62 group and the three contralateral uninjected sides are shown. The p62 antibody recognized both rat and human p62. Glyceraldehyde 3-phosphate dehydrogenase (GAPDH) was used for normalization. (TIF) [file pone.0169291.s002.tif]

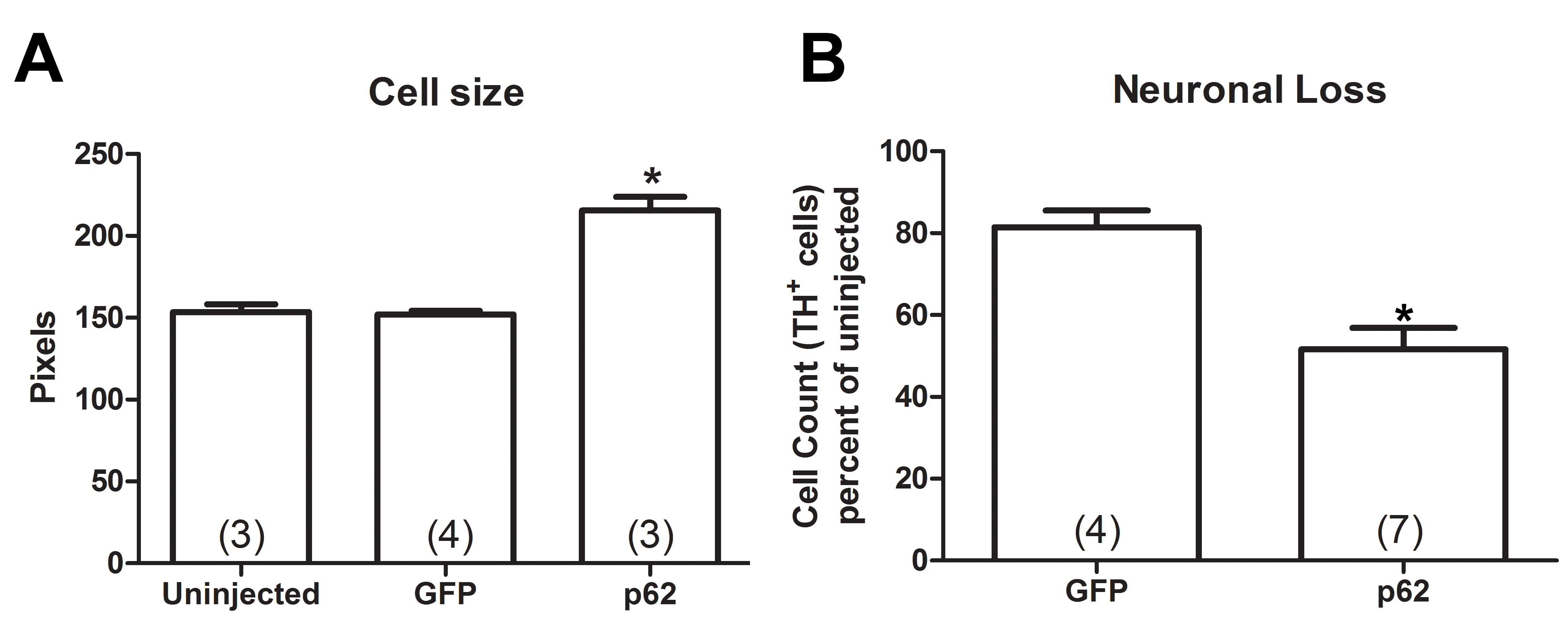

Supplement: S2 Fig — A) The tyrosine hydroxylase positive cells were significantly larger (41%) on the p62 side than the uninjected side (paired t-test, p < 0.05). There was no difference in cell size when AAV9 GFP was administered. B) Cells stained for tyrosine hydroxylase were counted by stereological analysis on the vector injected side and the uninjected side and expressed as a ratio for each animal. There was significant reduction of the tyrosine hydroxylase stained cells in the p62 group compared to the GFP group (t-test, p < 0.005). N values as indicated. (TIF) [file pone.0169291.s003.tif]

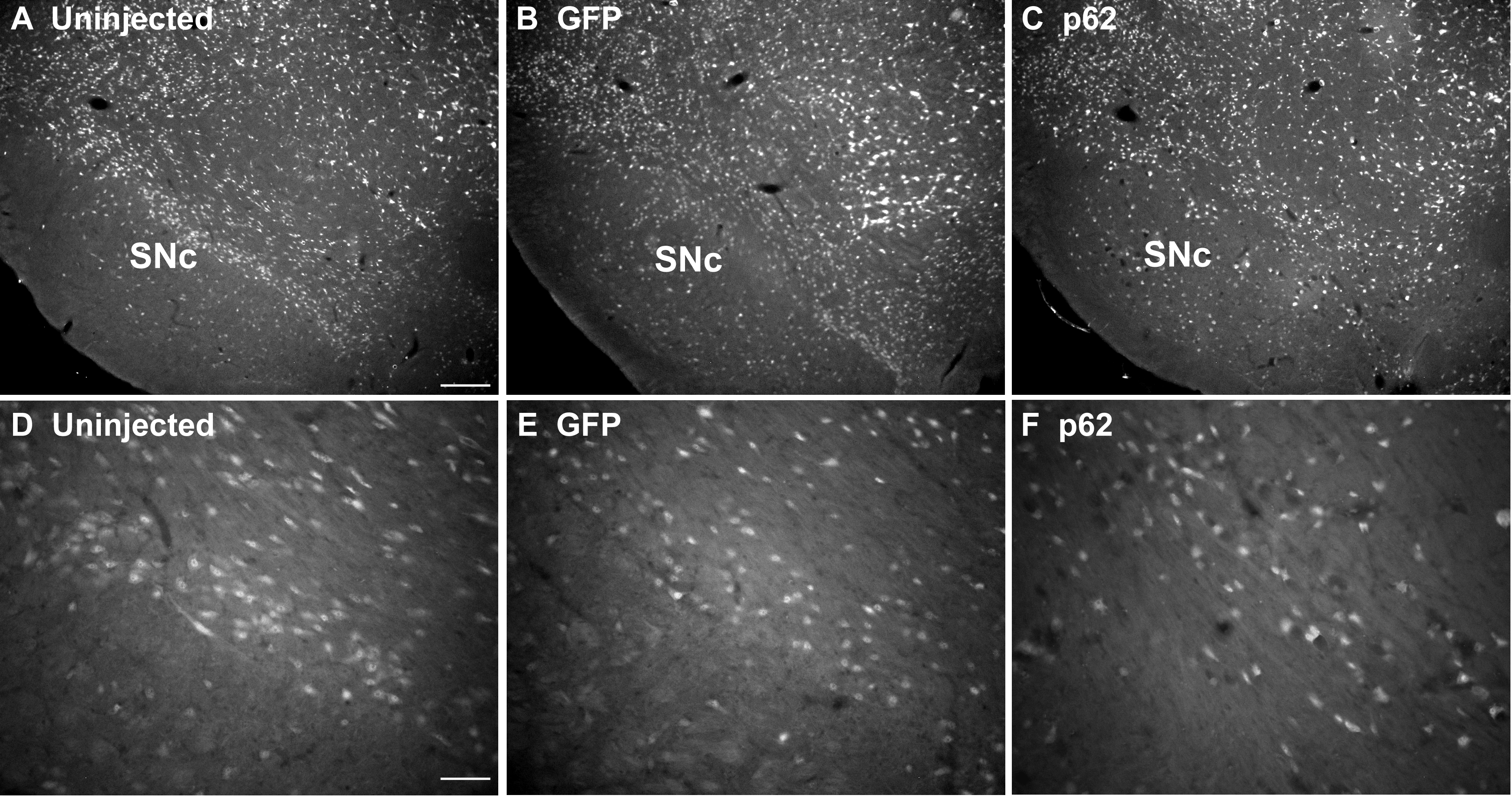

Supplement: S3 Fig — Compared to uninjected (A, D), there was evidence of a reduction in the number of stained cells in the substantia nigra pars compacta (SNc) in animals administered GFP (B, E) and p62 (C, F). Bar in A is 268 μm; same magnification in B and C. Bar in D is 67 μm; same magnification in E and F. (TIF) [file pone.0169291.s004.tif]

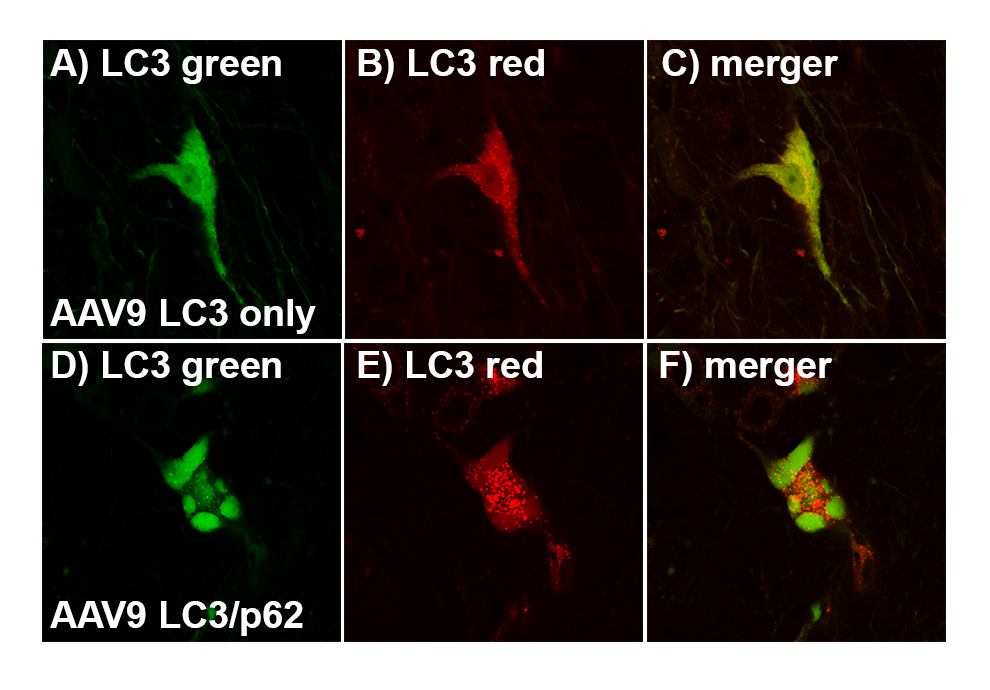

Supplement: S4 Fig — Confocal micrographs of neurons expressing double-tagged LC3, either alone (A-C) or with p62 (D-F). The p62 increased red-only puncta, consistent with the progression of LC3 to the autolysosome. The merged panels are also shown in Fig 3 with scale bars. (TIF) [file pone.0169291.s005.tif]

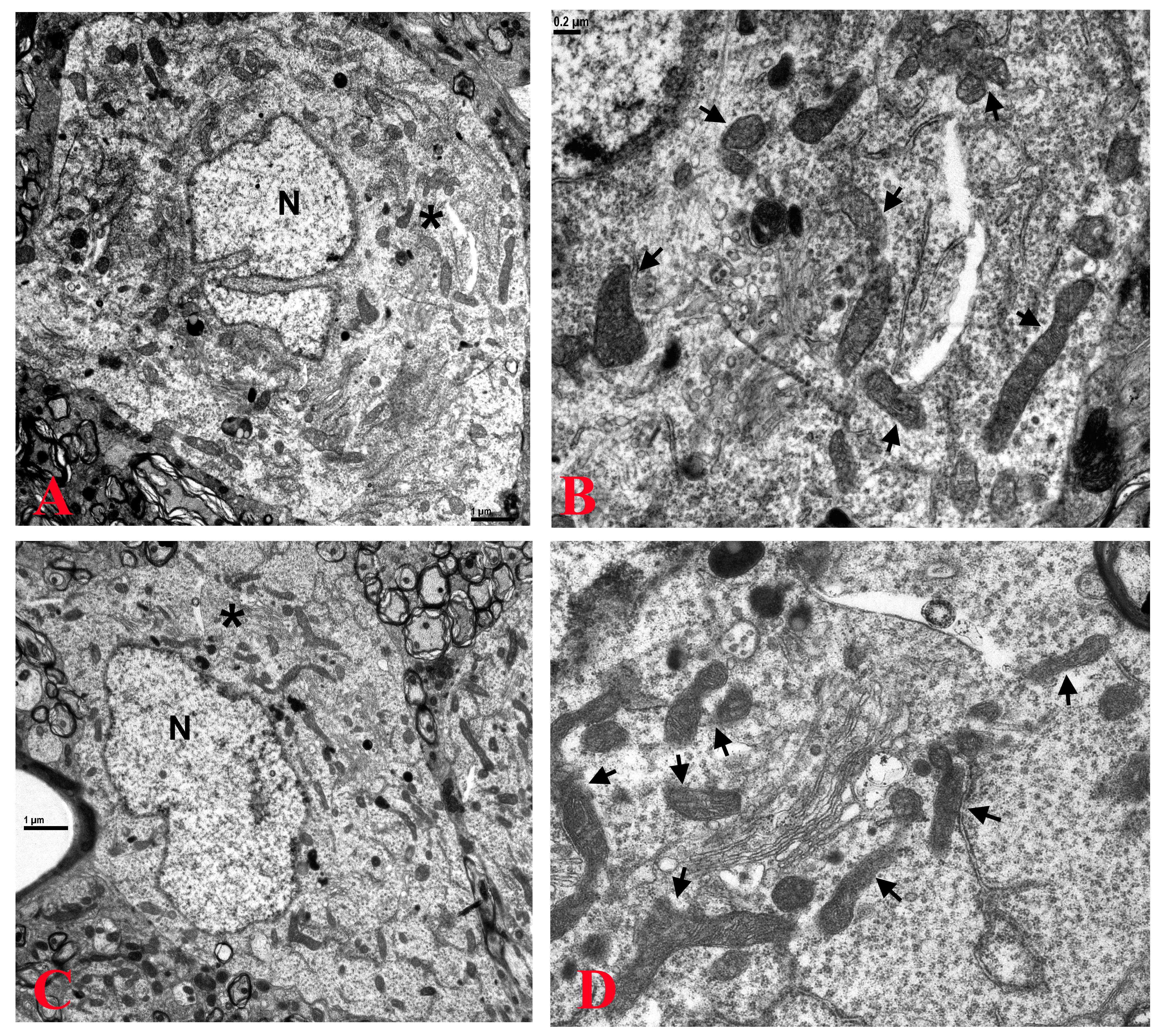

Supplement: S5 Fig — A, B) Neuron in the substantia nigra from the AAV9 GFP injected side. C, D) Neuron in the substantia nigra on the contralateral, uninjected side. B and D are enlargements of A and B, respectively. * in the left panels indicate area of enlargement on the right panels. Cytoplasmic inclusions of any size were absent. The mitochondria (arrows) did not contain vacuoles and there were few vesicles in the cytoplasm in contrast to the AAV9 p62 samples in Fig 5. N, nucleus. A patent vessel in C indicates a successful perfusion. Scale bars (1 μm) are shown in A and C. Scale bar (0.2 μm) is shown in B, same magnification in D. (TIF) [file pone.0169291.s006.tif]
